# Circulation of influenza A viruses among patients hospitalized for severe acute respiratory infection in a tertiary care hospital in Romania in the 2018/19 season: Results from an observational descriptive epidemiological study

**DOI:** 10.1097/MD.0000000000028460

**Published:** 2021-12-30

**Authors:** Anca Cristina Drăgănescu, Victor Daniel Miron, Anca Streinu-Cercel, Dragoş Florea, Ovidiu Vlaicu, Anuţa Bilaşco, Dan Oţelea, Monica Luminiţa Luminos, Daniela Piţigoi, Adrian Streinu-Cercel, Oana Săndulescu

**Affiliations:** a National Institute for Infectious Diseases “Prof. Dr. Matei Balş”, Bucharest, Romania; b Carol Davila University of Medicine and Pharmacy, Bucharest, Romania.

**Keywords:** clinical characteristics, hospitalization, influenza, vaccination

## Abstract

The seasonal circulation of influenza viruses and the impact that this infection has on the population varies from year to year. We have prospectively captured hospital-based surveillance data describing the circulation of influenza viruses and characterizing patients with influenza admitted to a tertiary hospital in Bucharest, Romania in the 2018/19 season.

We have conducted an observational descriptive epidemiological study analyzing all consecutive patients hospitalized for influenza like illness or severe acute respiratory infection at the National Institute for Infectious Diseases “Prof. Dr. Matei Balş”, Bucharest, Romania, from November 2018 to April 2019. For all patients we actively collected standardized clinical information and performed real-time reverse transcription polymerase chain reaction testing of respiratory samples to identify the presence of influenza viruses and to determine the subtype/lineage.

A total of 1128 hospitalized patients were tested in this study, with an influenza positivity rate of 41.2% (n = 465). We identified an exclusive circulation of influenza A viruses (A/H1 – 57.2%, A/H3 – 29.3%, A not subtyped – 13.3%), with only 1 case of influenza B detected at the end of the season (week 18/2019). Children under 5 years of age accounted for the majority of cases (40%, n = 186), and all cases had a favorable evolution. Females were more likely to test positive for influenza (53.3%) compared to males (46.7%), *P* = .048, and presence of asthma or chronic obstructive pulmonary disease increased the risk of influenza 4.4-fold and 2-fold, respectively (*P* < .001 and *P* = .034). Thirteen influenza patients required hospitalization in intensive care and 5 deaths were recorded (1.1%). The vaccination rate for all patients included in the study was low (4.6%). The existence of chronic conditions or age over 65 years prolonged the hospitalization period with 2 days (*P* < .001 each).

In the 2018/19 season, we identified an important circulation of influenza A viruses among patients hospitalized for influenza like illness/severe acute respiratory infection in a tertiary care hospital in Romania, with a higher likelihood of affecting females and patients with pre-existing lung conditions. Monitoring of the clinical and epidemiological characteristics of influenza virus infection is of great interest and should be done carefully each season to better inform on the necessary measures to limit the impact that this infection may have on risk groups.

## 1 Introduction

Influenza viruses have seasonal circulation with an increased incidence during the cold season (October–May for the Northern hemisphere). In humans, they infect the respiratory epithelium, the only site where the surface viral protein, hemagglutinin, is effectively cleaved, generating infectious virus particles.^[1]^ In addition to viral infectivity, host-related factors such as age, sex, chronic conditions, genetic predisposition, and microbiome play an important role in the clinical course and outcome of the disease.^[2]^

Although in most cases influenza is a self-limiting disease there are certain categories of patients^[3]^ – children under 5, elderly adults aged 65 and over, pregnant women and people with chronic conditions – who are at higher risk of severe complications of influenza, either bacterial superinfection or chronic disease decompensation, associating important morbidity and potentially leading to death. In these cases, influenza vaccination should be prioritized.^[4]^ Despite the fact that influenza vaccination is provided free of charge to people from risk groups in most countries including Romania,^[5]^ the overall vaccination rate remains suboptimal, well below the 75% threshold recommended for the elderly by the World Health Organization, with only 2 of the EU/EEA member states having reached this target, UK and the Netherlands, up to the 2014/15 influenza season.^[6]^

The circulation of influenza strains has been notoriously characterized as “predictably unpredictable”. For example, in Romania, there was a completely heterogeneous circulation in the 3 seasons that preceded the one reported in this study: in 2015/16 influenza A/H1 predominated,^[7]^ in 2016/17 influenza A/H3,^[8]^ and in 2017/18 influenza B circulated in more than two-thirds of cases.[^9^,^10^] For this reason, epidemiological surveillance is needed to detect temporal trends and to make informed decisions and projections regarding the upcoming influenza seasons. Under these conditions, laboratory confirmation of influenza, especially through molecular techniques, is important because it guides clinical therapeutic decisions. While surveillance data are widely available from Western European countries, less data are available for Eastern European countries, particularly Romania. Through our participation in international consortia for influenza epidemiological surveillance studies, we have prospectively captured hospital-based surveillance data.

The aim of this work is to describe the circulation of influenza viruses by type/subtype and characterize the outcomes of patients with influenza like illness (ILI)/severe acute respiratory infection (SARI) admitted to a tertiary hospital in Bucharest, Romania in the 2018/19 season.

## 2 Methods

### 2.1 Study design and study setting

In the 2018/19 influenza season, the National Institute for Infectious Diseases “Prof. Dr. Matei Balş” (NIID) was part of 2 international consortia for the surveillance of influenza: Global Influenza Hospital Surveillance Network (GIHSN)[^5^,^11^] and Development of Robust and Innovative Vaccine Effectiveness (DRIVE).^[12]^ Through our participation in these consortia, we conducted a prospective study based on active surveillance of cases of ILI or SARI, hospitalized in NIID, during the duration of the official national influenza surveillance for the season 2018/19, that is, from week 46/2018 to week 19/2019.

### 2.2 Study inclusion and exclusion criteria

The study methodology has been previously described in full.[^5^,^11^,^12^,^13^] In brief, we conducted an observational descriptive epidemiological study that included all consecutive patients who required hospitalization in NIID for 7-day onset ILI/SARI^[14]^ and who had an admission diagnosis compatible with a clinical condition used to identify admissions possibly associated with an influenza infection, as defined by the ICD 10 codes referenced by Puig-Barberà et al.^[11]^

Inclusion criteria:

- Patients hospitalized for 7-day onset ILI/SARI.
- Residents in the study catchment area from Romania: Bucharest-Ilfov, Dâmbovița, Giurgiu, Prahova, Argeş, Teleorman, Ialomița, Dolj, Vâlcea, Olt.
- Accepted to participate.
- Did not fulfill any of the exclusion criteria.

Exclusion criteria:

- Previously hospitalized in the 48 hours prior to symptom onset.
- Symptom onset ≥ 48 hours after admission to the hospital.
- Unwilling to participate or unable to communicate and give appropriate consent according to ethics regulations.
- Institutionalized at the time of symptoms onset.
- The respiratory specimen was taken ≥8 days after symptom onset.
- Tested positive for any influenza virus in the current season before the onset of symptoms leading to the current hospitalization.

### 2.3 Laboratory testing and clinical data collection

A commercial real-time reverse transcription polymerase chain reaction (RT-PCR) was used to detect the presence of influenza viruses or respiratory syncytial virus (RSV). Influenza positive samples were tested with a different rRT-PCR either to discriminate between influenza A H1 or H3 subtypes or between influenza B Yamagata or Victoria lineages, as previously described.^[5]^ Standardized clinical and epidemiological information was collected for each patient from their medical file and through direct interview, including details on the clinical presentation at admission, presence of pre-existing comorbid diseases, influenza vaccine status, and outcome of the current hospitalization.

### 2.4 Ethics approval

The protocols, the informed consent forms and the standardized case report forms were approved by the Bioethics Committee of the NIID – original approvals 9378/2018 and 9379/2018. Written informed consent was obtained before performing any study procedure from all adult patients, from the parents of minor children, or from the next-of-kin in the cases where the patient's severe clinical condition prohibited them from signing the informed consent themselves (i.e., patients receiving intensive care or mechanical ventilation at the moment of study inclusion).

### 2.5 Statistical analysis

The statistical analysis was performed with IBM SPSS Statistics for Windows, version 25 (IBM Corp., Armonk, NY). The level of statistical significance was set at *P* < .05. For continuous variables, we checked the normality of their distribution with the Shapiro-Wilk test. For variables that had normal distribution, we present the mean values and the standard deviation, and for the continuous variables with non-normal distribution we present the median and the interquartile range (IQR, defined as the 25^th^ and 75^th^ percentile), while the differences between the groups were analyzed with the Mann-Whitney *U* test and the Kruskal-Wallis H test. Effect size for the 2 tests was calculated as described in the literature.^[15]^ For categorical variables the frequencies and percentages are reported, and we used the chi-square test to calculate the odds ratio (OR) and 95% confidence interval (95%CI).

## 3 Results

### 3.1 Laboratory results

**Figure 1:**
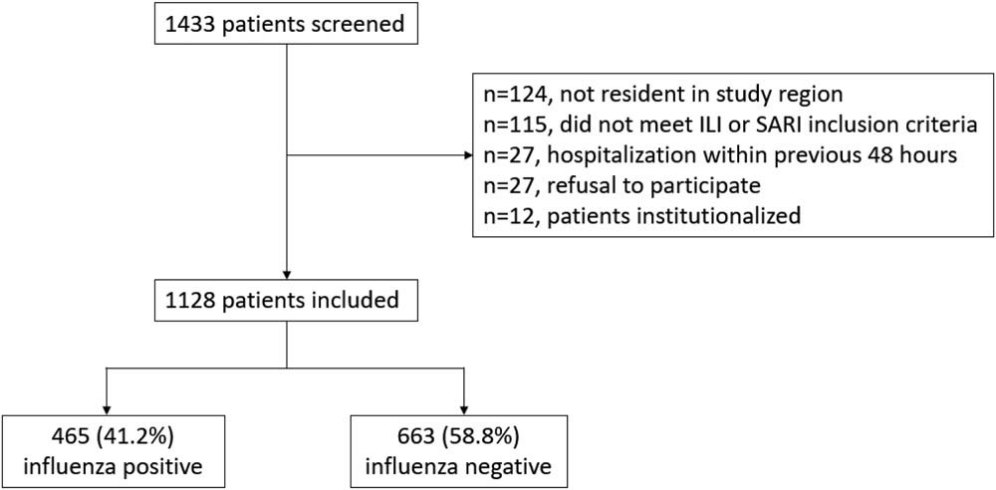
Patient flow chart. ILI = influenza like illness, SARI = severe acute respiratory infection.

During the study period, a total number of 1128 patients met the eligibility criteria and were tested for the presence of influenza viruses by RT-PCR (Fig. 1). The positivity rate for influenza was 41.2% (n = 465), while 96 patients (8.5%) tested positive for RSV and negative for influenza.

**Figure 2:**
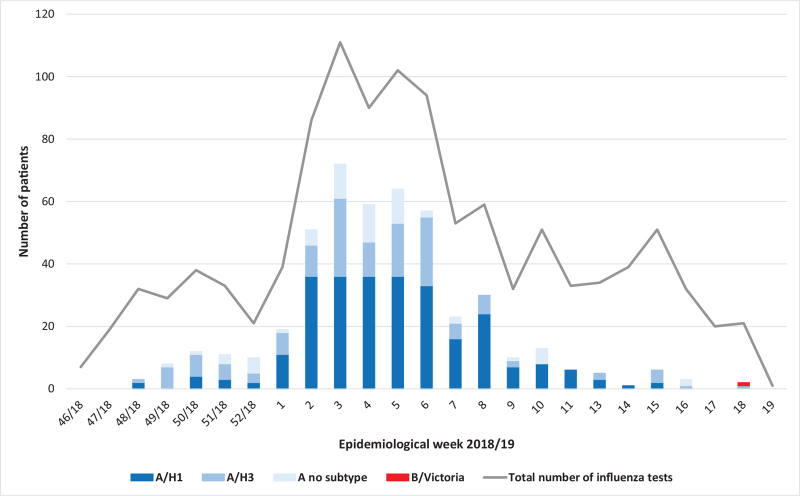
Distribution of viral subtypes by calendar week during the influenza season 2018/19.

The first case of influenza was identified in week 48/2018, and the last positive case was detected in week 18/2019. The highest rate of positivity was recorded in January and February 2019, weeks 2 to 6 (Fig. 2).

In this influenza season we identified an exclusive circulation of influenza A viruses (A/H1 – 57.2%, A/H3 – 29.3%, A not subtyped – 13.3%). Influenza A/H1 virus was identified as the main circulating subtype in all age groups (Table 1). Only 1 case of influenza B was detected, in week 18/2019, in a 45-month-old female preschooler, who had not been vaccinated against influenza, had no comorbidities, and was hospitalized for sudden onset fever, malaise, cough, and sore throat.

**Table 1:** Influenza positive patients by viral subtypes and age groups.

	Total patients tested, n (%)	Influenza positive, n (%)	Influenza A/H1, n (%)^∗^	Influenza A/H3, n (%)^∗^	Influenza A not subtyped, n (%)^∗^	Influenza B/Victoria^†^, n (%)^∗^
Infants, <1 yr	91 (8.1%)	48 (52.7%)	29 (60.4%)	17 (35.4%)	2 (4.2%)	0
Toddlers, 1–2 yrs	234 (20.7%)	81 (34.6%)	51 (63.0%)	26 (32.1%)	4 (4.9%)	0
Preschoolers, 3–4 yrs	144 (12.8%)	57 (39.6%)	42 (73.7%)	10 (17.5%)	4 (7.0%)	1 (1.8%)
School children, 5–13 yrs	124 (11.0%)	60 (48.4%)	39 (65.0%)	14 (23.3%)	7 (11.7%)	0
Teenagers, 14–17 yrs	16 (1.4%)	4 (25.0%)	2 (50.0%)	0 (0.0%)	2 (50.0%)	0
Young adults, 18–64 yrs	362 (32.1%)	144 (39.8%)	72 (50.0%)	43 (29.9%)	29 (20.1%)	0
Elderly adults, ≥65 yrs	157 (13.9%)	71 (45.2%)	31 (43.7%)	26 (36.6%)	14 (19.7%)	0
Total patients	1128 (100%)	465 (41.2%)	266 (57.2%)	136 (29.3%)	62 (13.3%)	1 (0.2%)

We detected no cases of influenza virus co-infection this season. However, 11 cases of influenza and RSV co-infection were identified – 9 cases A/H1 and 2 cases A/H3.

### 3.2 Characteristics and evolution of patients with laboratory-confirmed influenza

Compared with the laboratory-negative cases, there was a slight predominance of the female population among influenza-positive patients (*P* = .048) (Table 2). There were no statistically significant differences in median age between influenza positive [8 years (IQR: 2, 51)] and negative [9 years (IQR: 2, 51)] patients, *P* = .753. Fever, headache, myalgia, cough, and general deterioration were significantly associated with laboratory-confirmed influenza (LCI) – Table 2.

**Table 2:** Characteristics of patients tested.

	Total cases tested, n = 1128/N (%)	Influenza positive, n = 465/N (%)	Influenza negative, n = 663/N (%)	Statistical analysis
Gender				
Female	562 (49.8%)	248 (53.3%)	314 (47.4%)	***P* =** **.048**, χ^2^(1) = 3.9, OR = 0.8, 95%CI: 0.62–0.99
Male	566 (50.2%)	217 (46.7%)	349 (52.6%)	
Clinical presentations				
Fever	1069 (94.8%)	453 (97.4%)	616 (93.1%)	***P* =** **.001**, χ^2^(1) = 10.7, OR = 2.8, 95%CI: 1.48–5.38
Malaise	807 (71.5%)	341 (73.3%)	466 (70.4%)	*P* = .281
Headache	362 (32.1%)	182 (39.1%)	180 (27.2%)	***P* <** **.001**, χ^2^(1) = 17.9, OR = 1.7, 95%CI: 1.34–2.22
Myalgia	380 (33.7%)	186 (40.0%)	194 (29.3%)	***P* <** **.001**, χ^2^(1) = 14.0, OR = 1.6, 95%CI: 1.25–2.07
Cough	1012 (89.7%)	437 (94.0%)	575 (86.9%)	***P* <** **.001**, χ^2^(1) = 15.1, OR = 2.4, 95%CI: 1.52–3.68
Sore throat	392 (34.8%)	177 (38.1%)	215 (32.5%)	*P* = .053
Shortness of breath	283 (25.1%)	105 (22.6%)	178 (26.9%)	*P* = .101
Deterioration	524 (46.5%)	236 (50.8%)	288 (43.5%)	***P* =** **.016**, χ^2^(1) = 5.8, OR = 1.3, 95%CI: 1.06–1.70
Chronic conditions ≥1	485 (43.0%)	206 (44.3%)	279 (42.1%)	*P* = .472
Cardiovascular disease	197 (17.5%)	78 (16.8%)	119 (17.9%)	*P* = .601
COPD	36 (3.2%)	21 (4.5%)	15 (2.3%)	***P* =** **.034**, χ^2^(1) = 4.5, OR = 2.0, 95%CI: 1.04–4.00
Asthma	28 (2.5%)	21 (4.5%)	7 (1.1%)	***P* <** **.001**, χ^2^(1) = 14.0, OR = 4.4, 95%CI: 1.87–10.5
Diabetes	79 (7.0%)	40 (8.6%)	39 (5.9%)	*P* = .079
Immunodeficiency	72 (6.4%)	22 (4.7%)	50 (7.5%)	*P* = .057
Renal impairment	53 (4.7%)	19 (4.1%)	34 (5.1%)	*P* = .412
Rheumatologic disease	33 (2.9%)	16 (3.8%)	17 (2.6%)	*P* = .392
Neurological disease	37 (3.9%)	14 (3.4%)	30 (4.5%)	*P* = .194
Liver disease	70 (6.2%)	28 (6.0%)	42 (6.3%)	*P* = .825
Neoplasm	57 (5.1%)	21 (4.5%)	36 (5.4%)	*P* = .487
Obesity	94 (8.3%)	43 (9.2%)	51 (7.7%)	*P* = .356
Active tuberculosis	4 (0.4%)	2 (0.4%)	2 (0.3%)	*P* = .722
HIV infection	30 (2.7%)	11 (2.4%)	19 (2.9%)	*P* = .604
Influenza vaccination^∗^, season 2018–2019	52 (4.6%)	20 (4.3%)	32 (4.8%)	*P* = .675
Respiratory failure	137 (12.1%)	56 (12.0%)	81 (12.2%)	*P* = .930
ICU admission	35 (3.1%)	13 (2.8%)	22 (3.3%)	*P* = .620
Mechanical ventilation	10 (0.9%)	4 (0.9%)	6 (0.9%)	*P* = .938
Deaths	11 (1.0%)	5 (1.1%)	6 (0.9%)	*P* = .774

A percentage of 43.0% (n = 485) of the tested patients had at least 1 comorbidity. Cardiovascular disorders were the most common in both influenza-positive (16.8%) and influenza-negative (17.9%) patients. Chronic lung diseases such as chronic obstructive pulmonary disease (COPD) and asthma were associated with a 2.0, and respectively, 4.4-fold higher risk for influenza (Table 2). A percentage of 94.4% of elderly adults (≥65 years) with influenza had at least 1 associated comorbidity. Furthermore, the median (IQR) number of chronic conditions for this age group [3(2, 4)] was significantly higher (*P* < .001) compared with the rest of the influenza patients (Table 3). Median length of hospitalization for influenza positive patients was 5 days (IQR: 3, 7) (Table 3). The existence of chronic conditions [6 days (IQR: 4, 8) for patients with chronic conditions and 4 days of hospitalization (IQR: 3, 5) for patients without chronic conditions] or age over 65 years (Table 3) prolonged the hospitalization period with 2 days (*P* < .001 each).

**Table 3:** Characteristics of patients with influenza by age groups.

Characteristics	Infants, <1 yr, n = 48	Toddlers, 1–2 yrs, n = 82	Preschoolers, 3–4 yrs, n = 56	School children, 5–13 yrs, n = 60	Teenagers, 14–17 yrs, n = 4	Young adults, 18–64 yrs, n = 144	Elderly adults, ≥65 yrs, n = 71	Statistical analysis
Chronic conditions ≥1, n (%)	4 (8.3%)	14 (17.1%)	11 (19.6%)	10 (16.7%)	3 (75.0%)	97 (67.4%)	67 (94.4%)	N/A
Number of chronic conditions, median (IQR)	0 (0, 0)	0 (0, 0)	0 (0, 0)	0 (0, 0)	N/A^∗^	1 (0, 3)	3 (2, 4)	H = 238.345, ***P*** **<** **.001**, ε^2^ = 0.514
Born pre-term, n (%)^†^	2 (4.2%)	8 (9.8%)	2 (3.6%)	N/A	N/A	N/A	N/A	N/A
Days of hospitalization, median (IQR)	4 (3, 5)	4 (3, 6)	4 (3, 5)	4 (3, 5)	N/A^∗^	6 (3.25, 7)	7 (5, 10)	H = 75.073, ***P*** **<** **.001**, ε^2^ = 0.162
Influenza vaccination, n (%)^‡^								N/A
2018–2019 season	0	0	1 (1.8%)	3 (5.0%)	0	12 (8.3%)	4 (5.6%)	
2017–2018 season	0	0	0	1 (1.7%)	0	11 (7.6%)	3 (4.2%)	
2016–2017 season	0	0	0	1 (1.7%)	0	9 (6.3%)	2 (2.8%)	
RSV co-infection, n (%)	4 (8.3%)	5 (6.1%)	0	0	0	1 (0.7%)	1 (1.4%)	N/A
Respiratory failure, n (%)	2 (4.2%)	11 (13.4%)	0	2 (3.8%)	0	22 (15.3%)	19 (26.8%)	N/A
ICU admission, n (%)	1 (2.0%)	4 (4.9%)	0	1 (1.7%)	1 (25%)	4 (2.8%)	2 (2.8%)	N/A
Mechanical ventilation	0	0	0	0	0	2 (1.4%)	2 (2.8%)	N/A
Deaths, n (%)	0	0	0	0	0	3 (2.1%)	2 (2.8%)	N/A

Among the women hospitalized for ILI/SARI, 16 were pregnant. Ten (62.5%) of them tested positive for influenza (4 cases A/H1, 4 cases A/H3, 2 cases A not subtyped), and in 1 case RSV was identified. The majority of the pregnancies were of young gestational age (mean 19.5 ± 2.78 weeks, min. 10 weeks, max. 32 weeks). In all cases, the clinical evolution was favorable. Only 31.3% (n = 5) of all pregnant women had been vaccinated against influenza; among these, 2 had LCI.

Among children with ages below 5 years, we identified a number of 12 patients who had been born pre-term and who were positive for influenza (Table 3). We observed that pre-term children [median (IQR) of gestational age, 34.5 (33, 36) months] displayed an 11.2-fold increased risk of admission to the intensive care unit (ICU) [*P* = .002, χ^2^ = 9.39, OR = 11.2, 95%CI: 1.7–74.9] and a 5.4-fold increased risk of associating respiratory failure [*P* = .013, χ^2^ = 6.23, OR = 5.4, 95%CI: 1.3–23.0].

In case of patients with influenza-RSV co-infection, 9 (81.8%) of the 11 patients were children under 2 years (Table 3). The median duration of hospitalization was higher, but not statistically significant among patients with co-infection [6 days (IQR: 5, 10) vs. 5 days (IQR: 3, 7), *P* = .088]. We also observed that patients with co-infection had a 4.4-fold higher risk of respiratory failure [*P* = .012, χ^2^ = 6.3, OR = 4.4, 95%CI: 1.25–15.60] and a 16.6-fold higher risk of ICU admission [*P* < .001, χ^2^ = 24.8, OR = 16.6, 95%CI: 3.83–72.07] as compared with patients who were positive only for influenza.

A total of 13 (2.8%) patients with influenza required admission to the ICU (8 women and 5 men); 10 of them were adults with associated comorbidities, while the other 3 were children (12, 28 and 64 months, respectively) hospitalized with severe respiratory failure. None of the patients admitted to the ICU had been vaccinated against influenza. Of these, 4 required mechanical ventilation and 4 died, while the rest had a favorable evolution.

Among the patients confirmed with influenza there were 5 deaths (1%), all adults, 2 women and 3 men, none of them vaccinated against influenza:

- Woman, 26 years old (influenza A/H1), with hematological disease, hospitalized in the ICU and mechanically ventilated;
- Male, 37 years old (influenza A/H1) with cardiovascular disease, diabetes, renal disease, and obesity, hospitalized in the ICU and mechanically ventilated;
- Male, 40 years old (influenza A not subtyped), with HIV infection, cardiovascular disease, lung disease, neurological disease, and renal disease, hospitalized in the ICU;
- Woman, 76 years old (influenza A/H3) with cardiovascular disease, COPD, neurological disease, and obesity, hospitalized in the ICU and mechanically ventilated;
- Male, 93 years old, (influenza A/H3) with cardiovascular disease, diabetes, and renal disease, not hospitalized in the ICU.

### 3.3 Vaccination status

Overall, 4.6% (n = 52) of the patients included in the study had been vaccinated against influenza in the 2018/19 season, 48.1% of them (n = 25) with quadrivalent inactivated vaccine and 51.9% (n = 27) with trivalent inactivated vaccine. Of these, 53.8% (n = 28) had also been vaccinated in the previous 2 influenza seasons (2016/17 and 2017/18, respectively), and 5.8% (n = 3) only in the 2017/18 season. The vaccination rate was almost double in women (67.3%) as compared to men (32.7%). A number of 20 (38.5%) vaccinated patients (8 – quadrivalent vaccine and 12 – trivalent vaccine) were confirmed with influenza [9 – A/H1, 10 – A/H3, 1 – A not subtyped] (Tables 2 and 3), compared with 32 vaccinated patients (61.5%) who tested negative for influenza (*P* = .675). All vaccinated patients who had LCI had a favorable clinical evolution without complications.

## 4 Discussion

The current study has presented the circulation of different influenza virus types and subtypes, as well as the clinical and epidemiological characteristics of influenza in patients admitted to a tertiary hospital in Bucharest, Romania, in the 2018/19 season.

We identified an exclusive circulation of influenza A viruses (57.2% – A/H1, 29.3% – A/H3, and 13.3% – A not subtyped), and a single case of influenza B (0.2%), which is in line with the data communicated by the Romanian National Center for Surveillance and Control of Transmissible Diseases,^[9]^ which shows that during the 2018/19 season, only 1 case of influenza B, the one reported by us, was identified in Romania. Also, the European Center for Disease Prevention and Control^[16]^ showed that at European level, influenza A viruses have circulated in a proportion of 98.9%, with the predominance of the subtype A/H1 (57.4%), data confirming our report. This circulation is, however, different from the 2017/18 season when in Europe B-type influenza viruses circulated in a proportion of 64%.[^5^,^17^]

At the same time, the 2018/19 season was characterized by an early circulation of influenza viruses, with the first case of influenza being hospitalized in our institute in week 48/2018. An increased rate of positive influenza cases began in the weeks 2 to 3/2019, 3 weeks earlier than the 2017/18 season.^[9]^

It is well known that patients with chronic conditions are prone to complications of influenza and even death. In our study we identified an increased rate of comorbidities in patients with LCI (43.0%), the most frequent being cardiovascular disease. Similarly, Chung et al^[18]^ have identified high blood pressure and coronary heart disease as the main comorbidities in patients with influenza. On the other hand, San-Roman-Montero et al,^[19]^ in a 5-year study, have identified lung disease as the main category of comorbidities associated with influenza. In our study, we observed that patients with COPD and asthma hospitalized for ILI/SARI had a 2.0 and 4.4-fold higher risk of positivity for influenza, in line with the data seen from other studies.[^20^,^21^] Elderly adults aged 65 and over with influenza associated a significantly higher number of comorbidities compared to the other age groups and required longer hospitalization. Similar studies have found an increased risk of hospitalization among people ≥65 years of age with influenza, especially if they had at least 1 chronic condition.^[22]^ Overall, the elderly, especially those with chronic conditions or frailty, have a propensity for severe influenza outcomes. Moreover, elderly adults are vulnerable to diminished quality of life due to loss of independence following hospitalization with influenza.^[23]^ Therefore, public health policies must focus on protecting this group from such risks by increasing the vaccination rate and specific epidemiological measures during influenza seasons.

Pregnancy is another important risk factor for severe influenza-associated illness.^[24]^ In our study, we identified an influenza positive rate of 62.5% among pregnant women hospitalized for ILI. The evolution was favorable under medical supervision and treatment, in all cases. In fact, a meta-analysis of 36,498 pregnant women showed that pregnancy was associated with a 7-fold increased risk of hospitalization, but with a lower risk of admission to the ICU or death.^[25]^ Pregnant women represent a target group for vaccination prophylaxis, and any of the inactivated influenza vaccines can and should be safely administered in any trimester of pregnancy.[^26^,^27^] In the analyzed season, in Romania, only 0.1% (approx. 1300 doses) of influenza vaccines provided free by the Ministry of Health were administered to pregnant women.^[9]^ Increasing the vaccination rate in this segment of the population is particularly important, as it has been shown to have a double benefit on both the mother and the fetus. There are studies that have shown active transplacental transfer of postvaccine antibodies and no fetal vaccine-related adverse events have been reported.^[28]^ Thus, passive immunity can be provided to another risk group for influenza, infants under 6 months who are too young to receive influenza vaccination.

In the case of children under 5 years, we identified pre-term birth in the patient's history as a risk factor for admission to the ICU and the association of respiratory failure. Hardelid et al^[29]^ in a study of influenza in children with ages below 2 years, identified pre-term birth (<37 weeks) as a determinant of increased risk of hospital admission.

The timely identification of co-infections with other respiratory pathogens is very important, impacting both the patient's clinical management, and the patient's prognosis. There are numerous studies on bacterial superinfection of influenza, in particular for *Streptococcus pneumoniae*, and its associated impact on the evolution of the disease.[^30^,^31^] Viral co-infections can also be reasonably expected, given the overlap of the seasonality of the circulation of certain viruses. In our study we identified 11 (2.4%) cases of influenza-RSV co-infection. This value is comparable to another report for Romania (4.2%),^[5]^ but lower than the reports from Nepal (10.8%)^[32]^ and Madagascar (10.6%).^[33]^ Interestingly, patients with co-infection in our study exhibited an increased risk of respiratory failure and admission to the ICU compared to those with influenza without co-infection. These observations are significant and suggest the need to investigate a co-infection when hospitalizing a patient with influenza.

Influenza vaccination is available in Romania for all people over 6 months. For the 2018/19 season there were trivalent and quadrivalent inactivated influenza vaccines used in the country. The Ministry of Health provides, through general practitioners, free vaccination of people in groups at risk of developing severe influenza complications. However, the vaccination rate in the general population in Romania remains low. This aspect is observed in other countries, especially in those neighboring Romania.^[6]^ We reported in our study an overall low vaccination rate of 4.6%. At the national level, the Romanian National Center for Surveillance and Control of Transmissible Diseases reported a 6.8% uptake of the influenza vaccine in the general population and 20.9% in elderly patients.^[9]^ Although the percentages are low, they have somewhat increased as compared to the previous seasons: 5.2% vaccination rate for the 2017/18 season^[9]^ and 2.5% for the 2016/17 season.^[5]^ A percentage of 4.3% of the LCI cases had been vaccinated against influenza in the respective season. This rate may seem worrisome, however recent studies suggest that influenza vaccines were low to moderately effective for the 2018/19 season.[^34^,^35^] This may be hypothetically due to certain mismatches between the vaccine strain and the circulating virus. However, all cases of influenza in vaccinated patients displayed a favorable evolution, without complications, suggesting that some degree of protection did indeed exist.

At national level, in Romania, the mortality rate due to influenza in the 2018/19 season was reported at 8.6%,^[9]^ much higher than the one recorded in our study, where 1% of those with confirmed influenza died, all being adults with comorbidities. This reinforces the need for preventative health policies regarding the vaccination of people with chronic conditions.

Our study has aimed to provide a clear insight into the 2018/19 influenza season in Romania, an Eastern European country for which influenza surveillance data are limited. The large number of confirmed and hospitalized influenza cases, as well as the balanced breakdown by age groups of the patients included in the study provides a detailed picture of the circulation and impact of influenza in this region. The data presented are similar to other studies, but they contribute to the knowledge and characterization of seasonal variations of influenza depending on host factors, but also geographical and socio-economic factors. Our study had certain limitations: the selection of the study population was made from a single hospital, the NIID, which is also a reference center for infectious diseases in the southern region of Romania and beyond; this might have led to a large number of hospitalized influenza cases and thus a high rate of positivity among patients with ILI/SARI.

The variability of the circulation of influenza viruses from year to year underlines the necessity of continued monitoring of the impact that influenza has on the population, particularly among patients from influenza risk groups. Hence, in the long term, by comparing the data from year to year it will be possible to identify particular trends and to implement specific measures to limit the clinical impact of influenza in the population.

## 5 Conclusions

In this study we have reported an exclusive circulation of influenza A viruses in the 2018/19 influenza season, with the predominance of the A/H1 subtype throughout the season and in all age categories in a tertiary care hospital in Romania, with a higher likelihood of affecting females and patients with pre-existing lung conditions. In contrast to children, adults with influenza, especially those with chronic conditions and those aged 65 years and older, had more severe forms of illness that associated longer periods of hospital admission, ICU admission and, in some cases, death. Influenza surveillance remains a relevant topic, with extensive clinical and epidemiological studies needed each season to ensure consistent monitoring of the circulation of viral strains and the clinical impact that these may have on different risk groups.

## Author contributions

All authors had equal contributions.

**Conceptualization:** Anca Cristina Drăgănescu, Victor Daniel Miron, Daniela Pițigoi, Adrian Streinu-Cercel, Oana Săndulescu.

**Data curation:** Anca Cristina Drăgănescu, Victor Daniel Miron, Anca Streinu-Cercel, Daniela Pițigoi, Oana Săndulescu.

**Formal analysis:** Anca Cristina Drăgănescu, Victor Daniel Miron, Anca Streinu-Cercel, Dragoş Florea, Daniela Pițigoi, Oana Săndulescu.

**Funding acquisition:** Anca Cristina Drăgănescu, Victor Daniel Miron, Daniela Pițigoi, Oana Săndulescu.

**Investigation:** Anca Cristina Drăgănescu, Victor Daniel Miron, Anca Streinu-Cercel, Dragoş Florea, Ovidiu Vlaicu, Anuţa Bilaşco, Dan Oțelea, Monica Luminițta Luminos, Oana Săndulescu.

**Methodology:** Anca Cristina Drăgănescu, Victor Daniel Miron, Dragoş Florea, Ovidiu Vlaicu, Dan Oțelea, Daniela Pițigoi, Oana Săndulescu.

**Project administration:** Anca Cristina Drăgănescu, Victor Daniel Miron, Oana Săndulescu.

**Resources:** Anca Cristina Drăgănescu, Victor Daniel Miron, Dragoş Florea, Ovidiu Vlaicu, Dan Oțelea, Daniela Pițigoi, Oana Săndulescu.

**Software:** Anca Cristina Drăgănescu, Victor Daniel Miron, Dragoş Florea, Ovidiu Vlaicu, Oana Săndulescu.

**Supervision:** Anca Cristina Drăgănescu, Victor Daniel Miron, Dragoş Florea, Ovidiu Vlaicu, Dan Oțelea, Daniela Pițigoi, Oana Săndulescu.

**Validation:** Anca Cristina Drăgănescu, Victor Daniel Miron, Anca Streinu-Cercel, Dragoş Florea, Anuţa Bilaşco, Dan Oțelea, Monica Luminița Luminos, Oana Săndulescu.

**Visualization:** Anca Cristina Drăgănescu, Victor Daniel Miron, Anca Streinu-Cercel, Dragoş Florea, Oana Săndulescu.

**Writing – original draft:** Anca Cristina Drăgănescu, Victor Daniel Miron, Oana Săndulescu.

**Writing – review & editing:** Anca Cristina Drăgănescu, Victor Daniel Miron, Anca Streinu-Cercel, Dragoş Florea, Ovidiu Vlaicu, Anuța Bilaşco, Dan Oțelea, Monica Luminița Luminos, Daniela Pițigoi, Adrian Streinu-Cercel, Oana Săndulescu.
